# Intraperitoneal Rupture of the Urinary Bladder Mimics an Intra-Abdominal Hemorrhage: A Case Report

**DOI:** 10.7759/cureus.28275

**Published:** 2022-08-22

**Authors:** Kodai Shingaki, Tomohiro Abe, Tatsunori Ameda, Takeshi Nakamura

**Affiliations:** 1 Department of Surgery, Miyazaki Prefectural Miyazaki Hospital, Miyazaki, JPN; 2 Department of Emergency and Critical Care Medicine, University of Miyazaki, Miyazaki, JPN; 3 Department of Emergency Medicine, Miyazaki Prefectural Miyazaki Hospital, Miyazaki, JPN

**Keywords:** polytrauma, fracture, pelvis, laparotomy, focused assessment with sonography for trauma, hemorrhagic shock, rupture, urinary bladder

## Abstract

Hemorrhagic shock due to polytrauma is a life-threatening condition, requiring immediate diagnosis of the bleeding site and determination of an appropriate hemostatic procedure. Intra-abdominal injuries and pelvic fractures are major causes of massive hemorrhage, although the appropriate hemostatic procedures are different for each injury. We present a case of intraperitoneal rupture of the urinary bladder associated with pelvic fracture, in which urine extravasation into peritoneal spaces mimics intra-abdominal hemorrhage. A 33-year-old man with a known case of schizophrenia attempted suicide by jumping down from the 4th floor of his apartment (approximately 10 meters in height). He was in a state of shock on arrival. Focused assessment with sonography for trauma (FAST) showed fluid collection around his spleen only but not the perivesical space. Pelvic X-ray showed multiple pelvic fractures. We suspected the patient was in a state of hemorrhagic shock due to intra-abdominal hemorrhage and pelvic fracture. The patient's hemodynamic status did not respond to massive fluid infusion and blood transfusion, including eight units of packed RBCs transfusion. Resuscitative endovascular balloon occlusion of the aorta was performed; however, the patient's hemodynamic status did not recover. We performed an emergency laparotomy to control the suspected intra-abdominal hemorrhage. In peritoneal space, we found a large amount of non-bloody fluid. The liver, spleen, and bowels were not injured, whereas the urinary bladder was ruptured, indicating the correct diagnosis was intraperitoneal rupture of the urinary bladder associated with pelvic fracture. The ruptured urinary bladder wall was sutured, and temporary abdominal closure was performed. A contrast-enhanced CT performed after the laparotomy showed massive hemorrhage around the pelvic fracture. After arrival at the angiography room, the patient became bradycardia, and the pulsation at the carotid artery was not palpable. We performed cardiopulmonary resuscitation; however, the patient died eventually. Intraperitoneal rupture of the urinary bladder would mimic an intra-abdominal hemorrhage. Therefore, a comprehensive diagnostic-treatment approach such as a hybrid ER system would be beneficial for early and accurate diagnosis.

## Introduction

Trauma is one of the major causes of death all over the world. Hemorrhage counts for over 30% of all-cause trauma mortality, though it is a treatable cause of death [1]. Rapid identification of the bleeding site, resuscitation with massive fluid and blood transfusion, and hemostasis are essential strategies for treating trauma patients in a state of hemorrhagic shock [2]. Surgical hemostasis and transcatheter arterial embolization (TAE) are two major hemostatic procedures to control hemorrhage. TAE has played the primary role in controlling arterial bleeding from sites difficult to being approached surgically and endoscopically, such as pelvic fractures [3]. Surgical hemostasis is accepted as an important hemostatic procedure even in the modern era with developed TAE procedures. Particularly, massive abdominal hemorrhage often requires an immediate surgical hemostatic approach because it often cannot be controlled by TAE [4]. Clinicians, therefore, should determine the most appropriate hemostatic approach according to the bleeding sites. Focused assessment sonography for trauma (FAST) is a rapid and useful examination for trauma patients [5]. FAST has a diagnostic accuracy of over 95% for 200 mL intra-abdominal fluid collection [6] and has 83% specificity for therapeutic laparotomy in hemodynamically unstable trauma patients [7]. Traumatic urinary bladder rupture is often associated with pelvic fracture, which occurs in 5-10% of all pelvic fractures [8]. The urinary bladder rupture is classified as intraperitoneal rupture, extraperitoneal rupture, and mixed rupture, according to the range of urinary leakage [9]. Intraperitoneal rupture is when urine leaks into the peritoneal cavity. We herein report a case of intraperitoneal rupture of the urinary bladder, which mimics massive intra-abdominal hemorrhage through the FAST. Hemostatic laparotomy was performed for suspected intra-abdominal hemorrhage, although the major source of bleeding was the pelvic fracture. In hemodynamically unstable polytrauma patients, intraperitoneal rupture of the urinary bladder is not a cause of circulatory failure but may induce critical mistakes in clinical decisions.

## Case presentation

**Figure 1:**
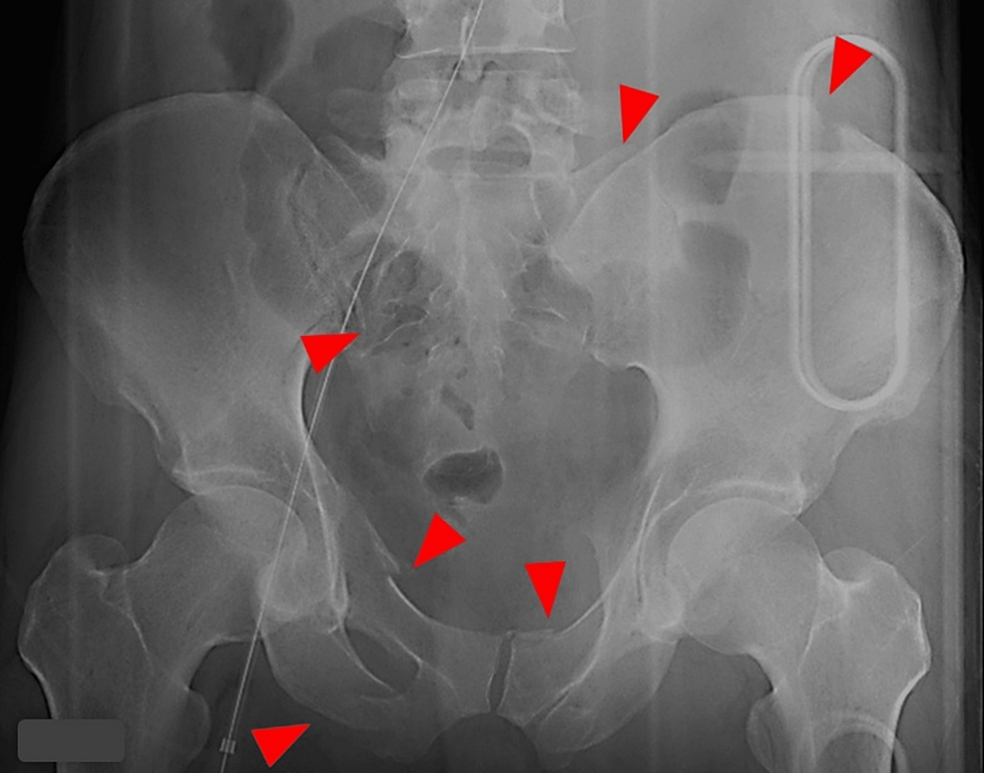
Pelvis X-ray. Multiple fractures (arrowheads) are found on both sides of the pelvis on the X-ray.

A 33-year-old man under treatment with oral medications for schizophrenia jumped off the 4th floor of his apartment (approximately 10 meters in height) in a suicide attempt. He received a peripheral cannula insertion prehospitally, and he was transported by ambulance to an ER at the Miyazaki Prefectural Miyazaki Hospital. On arrival, the patient's heart rate was 140 per minute, blood pressure was unmeasurable, and the patient's radial pulse was poorly palpable. In addition, the patient's oxygen saturation was unmeasurable. The patient's Glasgow Coma Scale was 8 (E1V2M5), pupils showed anisocoria at 3 mm/ 4mm (right/left), and pupillary reflexes to light were sluggish. FAST showed obvious fluid collection around the spleen, whereas it was obscure in perivesical space. Pelvic X-ray showed multiple pelvic fractures (Figure 1).

Blood laboratory tests showed elevations of urea nitrogen, creatinine, liver enzymes, fibrin degradation products, and d-dimer. Blood gas analysis showed acidosis and markedly elevation of lactate (pH 7.113, lactate 7.5 mmol/L). On admission, the levels of hemoglobin were not decreased yet (Table 1).

**Table 1:** Laboratory values on arrival.

Variables	Results	Normal range
WBC count (×10^3^/μL)	12.0	8.6-3.3
Hemoglobin (g/dL)	15.3	13.7-16.8
Platelet count (×10^4^/μL)	393	158-348
Urea nitrogen (mg/dL)	30.2	8.0-20.0
Creatinine (mg/dL)	3.04	0.65-1.07
Aspartate aminotransferase (IU/L)	107	13-30
Alanine aminotransferase (IU/L)	66	38-113
Lactate dehydrogenase (IU/L)	1149	124-222
C-reactive protein (mg/dL)	0.29	0.00-0.14
Prothrombin time (%)	65.4	70.0-130.0
Prothrombin time international normalized ratio (INR)	1.26	
Activated partial thromboplastin time (sec.)	41.9	24.0-39.0
Fibrinogen (mg/dL)	248	200.0-400.0
D-Dimer (μg/mL)	70.5	0.00-1.00
Fibrin degradation product (μg/mL)	603	0.0-5.0
(Arterial gas analysis)		
pH	7.113	7.350-7.450
pCO2 (mmHg)	49.9	35.0-45.0
pO2 (mmHg)	600	75.0-110.0
HCO3 (mEq/L)	15.3	20.0-26.0
Base excess (mEq/L)	-14.4	-3.0-3.0
Lactate (mmol/L)	7.5	0.50-2.00

**Figure 2:**
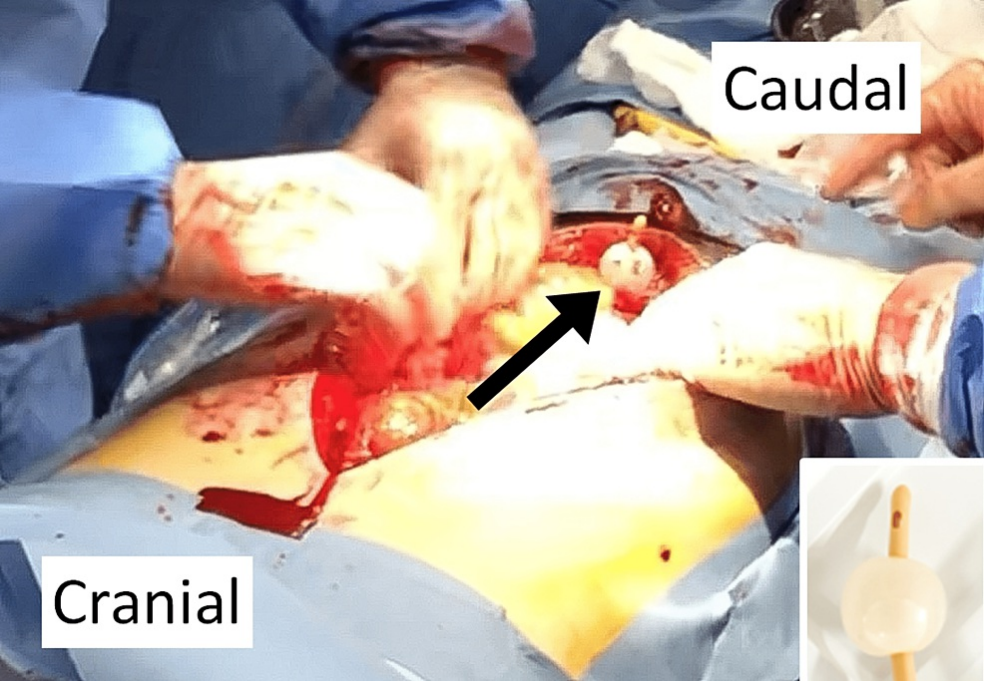
Findings during laparotomy. The bladder balloon (black arrow) can be seen from in the peritoneal space, indicating an intraperitoneal rupture of the urinary bladder. Right-bottom panel shows the same bladder balloon.

We diagnosed the patient in a state of hemorrhagic shock due to intra-abdominal hemorrhage and pelvic fracture, and we started massive fluid infusion and blood transfusions. We performed tracheal intubation to secure the airway because the patient was in a severe hemorrhagic shock and needed surgical hemostasis and transarterial embolization. Resuscitative endovascular balloon occlusion of the aorta (REBOA) was inserted from the right femoral artery because the patient's circulatory function could not be stabilized with eight units of packed RBC transfusion. In addition, the patient's hemodynamics were unstable even after resuscitation with the REBOA. We, therefore, decided to perform hemostasis by emergency laparotomy before performing a CT scan. The patient underwent a crash laparotomy [10]. Through the survey of the abdominal cavity, there were no clots, no organ damage, and no active bleeding in the abdominal cavity. At the same time, there was serous fluid in the abdominal cavity. A tear was observed at the apex of the bladder, and a urinary catheter was exposed in the abdominal cavity (Figure 2).

**Figure 3:**
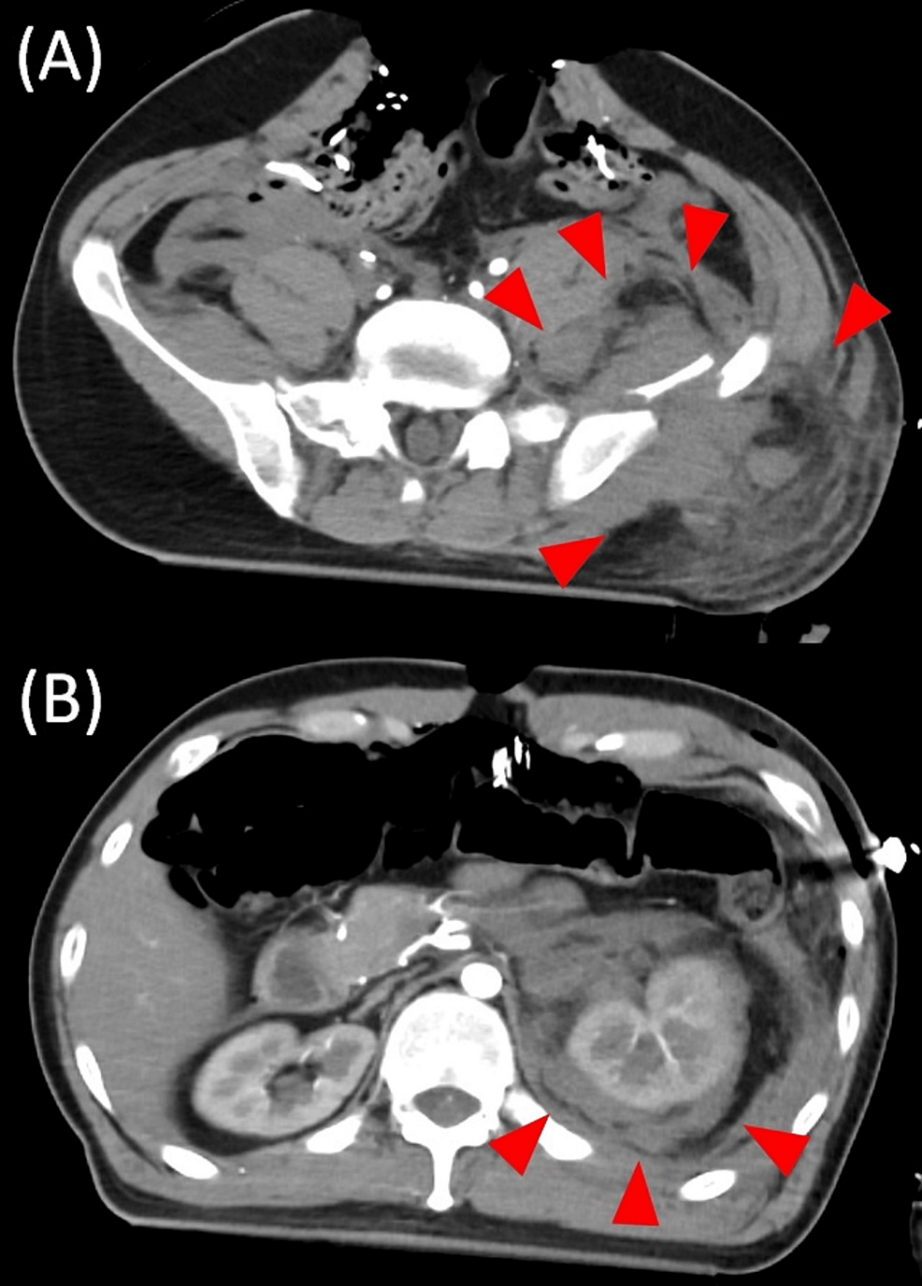
CT of the pelvis and abdomen. (A) CT showed obvious hematoma (arrowheads) from around the pelvic fracture to (B) the perirenal space.

We diagnosed that the fluid collection detected by FAST was urine leaked from the ruptured bladder. There was swelling around the bladder, while there was no expanding or pulsatile hematoma around the kidneys. When the anterior bladder cavity was peeled off, bleeding around the prostate occurred, so we packed it with gauzes. Next, we sutured the ruptured bladder and closed the abdomen using Baker's method. The CT scan of the head showed traumatic subarachnoid hemorrhage and marked cerebral edema, indicating diffuse axonal injury. Abdominal and pelvic CT showed a large hematoma from multiple pelvis fractures to the left kidney. Contrast-enhanced CT scan showed no bleeding sites other than around the pelvic fracture, indicating the most critical source of bleeding (Figure 3).

The patient's injury severities were diagnosed as follows: injury severity score of 43, revised trauma score of 4.415, and the probability of survival of 0.384. We decided to perform transarterial embolization and transported the patient to the angiography room. Before vascular sheath insertion, the patient's heartbeat became bradycardic, and the pulsation at the carotid artery became not palpable. The patient's spontaneous circulation did not return despite immediate cardiopulmonary resuscitation, and the patient died eventually.

## Discussion

We presented a case of intraperitoneal rupture of the urinary bladder caused by a pelvic fracture. Because the patient was in shock, we misdiagnosed the urine in the abdominal cavity as intra-abdominal hemorrhage and performed an emergency laparotomy. Unfortunately, the patient died before performing TAE for pelvic fracture eventually. Patients with urinary bladder rupture generally show abdominal pain, hematuria, and increased creatinine levels. However, these signs may not be obtained from a critically injured patient requiring immediate surgery. Contrast-enhanced CT scan is not useful for diagnosing urinary bladder rupture [11]. CT cystography is a diagnostic CT scan procedure using contrast media injection to the urinary bladder via a catheter. It has 95% sensitivity and 100% specificity for diagnosing urinary bladder rupture [12], although it requires an adequate volume of media (at least 350 mL of diluted media) and a certain procedural time [13]. The diagnostic peritoneal lavage (DPL) would distinguish intraperitoneal urine from the blood. However, it is not indicated for hemodynamically unstable blunt trauma patients showing positive abdominal FAST results [14]. Thus, the accurate diagnosis of intraperitoneal rupture of the urinary bladder is difficult in hemodynamically unstable patients. Similar to our case, two cases of intraperitoneal rupture of the urinary bladder have been reported [15]. Both cases were hemodynamically unstable and showed positive results of abdominal FAST despite the absence of intra-abdominal hemorrhage. They underwent laparotomy prior to the TAE for bleeding around the pelvic fracture. Because emergency laparotomy is effective in hemodynamically unstable intra-abdominal hemorrhage patients [16], clinicians would determine emergency laparotomy in a hemodynamically unstable trauma patient with a sign of intra-abdominal hemorrhage. Therefore, intraperitoneal rupture of the urinary bladder mimics a massive intra-abdominal hemorrhage through FAST. It also urges clinicians to determine immediate laparotomy. Even though the intraperitoneal rupture of the urinary bladder requires surgical repair, clinicians might determine the wrong strategy. In our case, the whole-body CT scan was performed after the laparotomy because the patient's hemodynamics were so unstable to transport to the remote CT room. Rapid and accurate diagnosis of the most critical injury is challenging in hemodynamically unstable patients. The immediate total-body CT scan protocol would be a better practice, although it did not show a reduction in the mortality of patients with severe trauma [17]. The most effective clinical algorithm would depend on each institutional environmental factor. A hybrid ER system would have merit for improving clinical practice for severe trauma patients. This comprehensive diagnostic-treatment system allows rapid and accurate diagnosis and treatment [18]. Clinicians can provide initial resuscitation, CT scan, and perform surgical hemostasis and TAE without transporting the patient from the room. Because intraperitoneal rupture of the urinary bladder is the most deceiving situation, the hybrid ER system would allow us to determine the correct diagnosis and treatment strategy. 

## Conclusions

We presented a case of intraperitoneal rupture of the urinary bladder associated with pelvic fracture, which was misdiagnosed as massive intra-abdominal hemorrhage. Intraperitoneal rupture of the urinary bladder would mimic a massive intra-abdominal hemorrhage. It may result in a wrong emergency laparotomy for hemostasis, especially in a hemodynamically unstable patient due to hemorrhage from pelvic fractures. Comprehensive diagnostic-interventional approaches, such as a hybrid ER system, would help clinicians to diagnose and provide treatments correctly.
